# Circle of care model for strengthening newborn care through Kangaroo Mother Care integration in primary health systems in rural India

**DOI:** 10.1136/bmjpo-2026-004568

**Published:** 2026-09-03

**Authors:** Yogita Thakral, Ashmita Gulechha, Sanjana Brahmawar Mohan, Pavitra Mohan

**Affiliations:** 1 Basic HealthCare Services, Udaipur, India

**Keywords:** Child Health, Technology, Nursing Care, Low and Middle Income Countries

## Abstract

**Background:**

Kangaroo Mother Care (KMC) reduces morbidity and mortality among preterm and low birth weight infants, yet continuity between facility initiation and home practice remains weak in rural settings. This study examined a facility-community circle of care model in a primary care setting in southern Rajasthan, India.

**Methods:**

In a primary health centre (PHC) managed as a public–private partnership in rural south Rajasthan, India, we trained and supported the PHC facility-based team to initiate KMC for all low birth weight babies at birth, and the outreach team to continue KMC at household levels. Both teams educated and supported mothers and family members to provide and sustain KMC for the babies.

We reviewed the programme and supervisory data of a 3-year period (2021–2024) following the KMC introduction to estimate key indicators of its coverage and effectiveness. We also conducted semi-structured interviews with the team members to understand and document the process of introduction.

**Results:**

Among 1188 live births, 34% were low birth weight. Facility KMC coverage rose from 60% to 88%, and 52% of dyads continued KMC at home. Exclusive breastfeeding reached 98%, mean skin-to-skin contact averaged 9 hours daily and infant survival was 99%. The circle of care strengthened continuity, trust and workforce capability.

**Conclusion:**

Embedding KMC within a coordinated, mentorship-supported circle of care provides a scalable systems approach to enhancing newborn outcomes and primary care in rural and tribal settings.

**WHAT IS ALREADY KNOWN ON THIS TOPIC**

- Kangaroo Mother Care (KMC) is a proven, low-cost intervention that reduces morbidity and mortality among preterm and low birth weight infants, but most evidence focuses on facility-based implementation with limited documentation of continuity after discharge, especially in rural, tribal and migratory settings.

**WHAT THIS STUDY ADDS**

- This study shows that a primary healthcare-community ‘circle of care’, initiated during antenatal care and sustained through postnatal home follow-up, can successfully integrate facility-based and community-based KMC, strengthen frontline workforce capacity and enable families to act as co-creators of newborn care.

**HOW THIS STUDY MIGHT AFFECT RESEARCH, PRACTICE OR POLICY**

- The findings suggest that embedding KMC within horizontally integrated primary health centres-community systems, supported by field-based mentorship and family participation, can improve continuity, equity and trust in newborn care, and could inform the design of scalable newborn health strategies in low-resource and mobile populations.

## Introduction

 Kangaroo Mother Care (KMC) is an evidence-based, cost-effective intervention proven to reduce morbidity and mortality among preterm and low birth weight infants.^1 2^ KMC comprises prolonged skin-to-skin contact, support for exclusive breastfeeding, early discharge when clinically appropriate and structured post-discharge follow-up. Existing evidence from both facility-based and community-based studies has consistently demonstrated its effectiveness in improving neonatal survival, breastfeeding outcomes, thermal regulation and early newborn recovery.^3–5^ The immediate KMC trial highlighted that initiation of KMC before full clinical stabilisation reduced neonatal mortality among small and preterm newborns, contributing to revised global recommendations supporting early initiation of KMC.^6^

Despite this well-established evidence base, operationalising and sustaining KMC within routine primary healthcare systems remains challenging, particularly in rural and resource-constrained settings. Existing literature has predominantly focused on clinical outcomes, neonatal unit implementation or short-term facility-based delivery of KMC.^7^ Limited attention has been paid to how KMC can function as a longitudinal ‘circle of care’ that links antenatal counselling, facility-based initiation, post-discharge follow-up and community-based newborn care across the maternal-newborn continuum. This gap is especially important in rural, tribal and migratory populations where continuity between facilities and households is often disrupted by geographical barriers, workforce shortages, migration, transportation constraints and weak referral linkages.^8–10^

In India, primary health centres (PHCs) are the first point of contact for maternal and newborn care in rural areas. Despite increased institutional deliveries, continuity of newborn care after discharge remains inconsistent, especially for vulnerable low birth weight (LBW) and preterm infants. Although Accredited Social Health Activists (ASHAs) and Auxiliary Nurse Midwives (ANMs) support postnatal follow-up, integration between facility-based KMC initiation and home continuation remains poorly documented within routine PHC systems.

Existing evidence suggests that frontline mentorship, supportive supervision, counselling reinforcement and family participation are important for sustaining KMC after discharge.^11 12^ However, less is known about how these elements can be operationally integrated within routine PHC systems to sustain continuity of care across antenatal, facility and household settings in rural and migratory populations.

The objective of this work is to integrate KMC and management across facility and community settings of a functional primary healthcare system, and to develop and document an integrated maternal-newborn circle of care model for remote, rural, tribal and migratory settings.

This paper describes the operationalisation of a mentorship-driven PHC-community KMC circle of care within a public–private partnership (PPP) primary healthcare model in a rural, tribal and migratory region of southern Rajasthan, India. It provides operational insights into how continuity of newborn care was sustained across antenatal, facility and household settings through coordinated engagement of PHC nurses, ANMs, ASHAs and families within a routine rural primary healthcare system.

## Materials and methods

### Study setting

This paper presents an implementation assessment using routine programme data from a PHC operating under a PPP model in Nithauwa, Dungarpur district, Rajasthan, India. The PHC serves approximately 25 000 people across 23 predominantly tribal villages characterised by dispersed settlements, seasonal migration, poor road connectivity and limited access to referral care.

In 2015, the Government of Rajasthan partnered with Basic HealthCare Services (BHS), a non-profit organisation, to strengthen service delivery at the PHC. Under this arrangement, the government provided infrastructure, medicines, consumables and community-level incentives, while BHS managed staffing, service delivery, supervision and quality assurance. The PHC team included a medical officer, nurses, a pharmacist, laboratory technicians and six outreach subcentres staffed by ANMs and ASHAs.

KMC was introduced into routine maternal and newborn care in September 2021.

### Development of the integrated newborn care model

The intervention was developed in response to operational challenges in maintaining continuity of care for LBW and preterm newborns across facility and home settings. Prior to implementation, a formative phase was conducted between April and August 2021 involving consultations with PHC nurses, ANMs, ASHAs, supervisors and mothers accessing antenatal and postnatal services.

The formative assessment identified several barriers including limited familiarity with prolonged skin-to-skin care, inconsistent continuation of KMC following discharge, difficulties in identifying and following up vulnerable newborns, geographical barriers, seasonal migration and weak coordination between facility and community health workers.

Based on these findings, an integrated newborn care model was developed linking antenatal counselling, facility-based KMC initiation, community-based continuation of KMC, structured newborn follow-up and referral support. During the first 6 months of implementation, supervisory reviews and field visits were used to refine counselling approaches, follow-up schedules, referral pathways, documentation systems and supportive supervision processes.

### Intervention

The intervention integrated antenatal counselling, facility-based KMC initiation, post-discharge community follow-up and referral support within routine maternal and newborn care services. The model was developed in response to challenges in maintaining continuity of care for LBW and preterm newborns in remote communities characterised by geographical barriers, migration and limited referral access.

PHC nurses, ANMs and ASHAs received training in the identification and management of LBW newborns, KMC practices, breastfeeding support, newborn danger signs and family counselling. Ongoing mentorship and supportive supervision were provided through joint field visits, case reviews and regular supervisory meetings.

**Figure 1:**
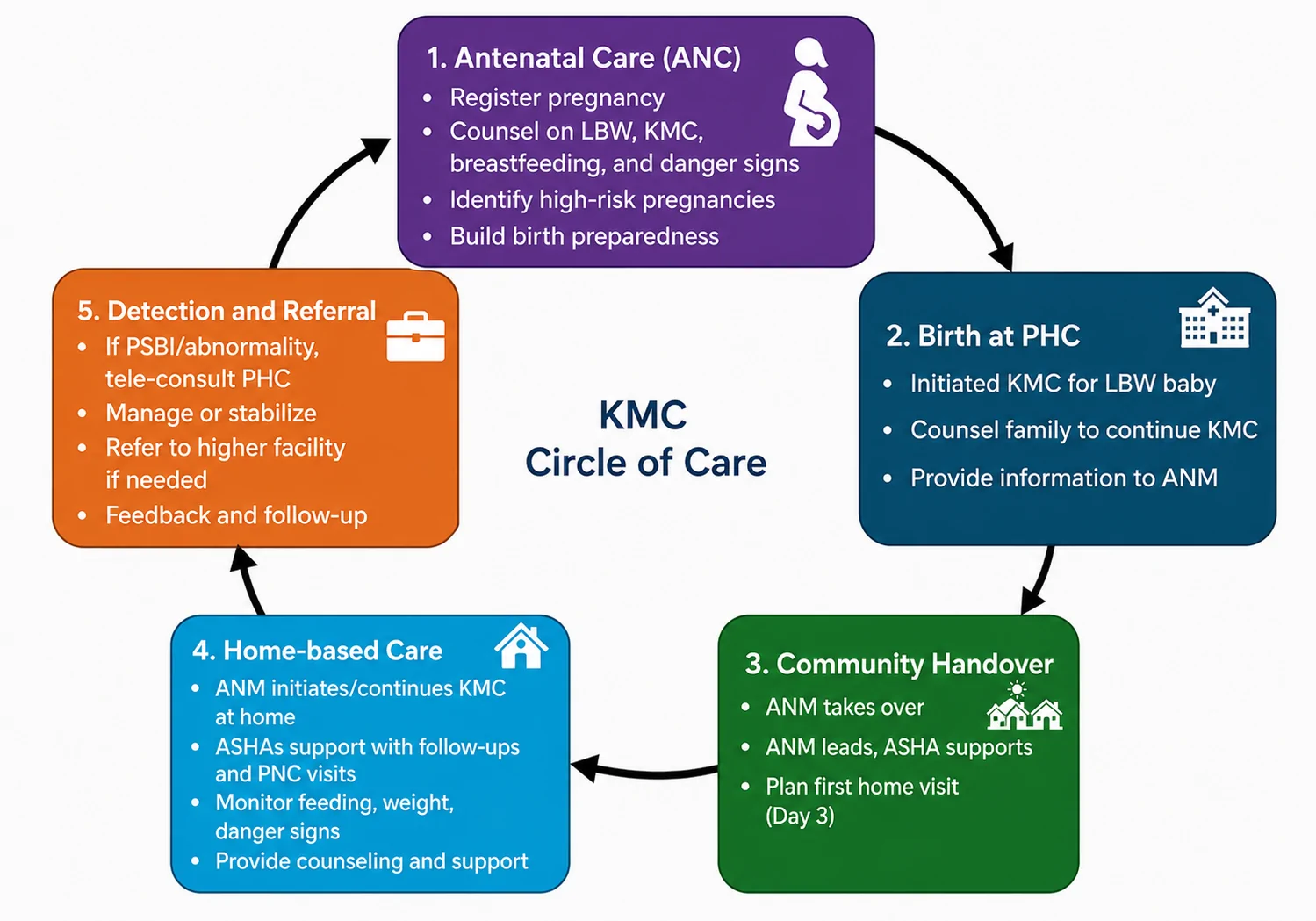
Intervention details circle of care. ANM, Auxiliary Nurse Midwives; ASHAs, Accredited Social Health Activists; KMC, Kangaroo Mother Care; LBW, low birth weight; PHC, primary health centre; PNC, postnatal care; PSBI, possible serious bacterial infection.

Clinically stable LBW newborns were initiated on KMC shortly after birth. Mothers and family members received counselling on skin-to-skin care, exclusive breastfeeding, thermal care and newborn danger signs. Following discharge, ANMs conducted home visits on day 3, day 7 and weekly thereafter for up to 8 weeks to support continuation of KMC, monitor infant growth and feeding, identify illness and facilitate referral where necessary. ASHAs provided additional household-level follow-up and counselling support. This circle of care is depicted in figure 1:

All live births occurring at PHC Nithauwa between September 2021 and May 2024 were included in the assessment. The intervention primarily targeted LBW newborns (<2500 g) and clinically stable preterm infants. Follow-up support was provided through facility and community-based services, with continuity of care maintained through coordination between PHC staff, ANMs and ASHAs.

Newborns with very LBW or those requiring intensive neonatal care or higher-level stabilisation were referred to tertiary facilities. Implementation quality was monitored through supportive supervision, direct observation of KMC practices, review of counselling sessions, verification of follow-up records and monthly review meetings. Supervisory assessments were conducted by senior nursing staff, programme coordinators and PHC leadership teams.

Implementation reviews were used to identify operational bottlenecks and inform adaptations to counselling approaches, follow-up schedules, referral pathways and documentation practices.

### Data sources, process indicators and outcome measures

The assessment used routinely collected programme and supervisory data recorded between September 2021 and May 2024. Data sources included facility delivery registers, newborn care registers, KMC monitoring records, outreach follow-up registers and supervisory documentation.

Quantitative indicators included live births, LBW newborns, KMC initiation, continuation of KMC after discharge, breastfeeding practices, postnatal follow-up visits, identification and referral of newborn danger signs and newborn outcomes during follow-up. Qualitative data were obtained from structured field notes documented during supervisory visits, mentorship interactions, implementation review meetings and semi-structured interviews with PHC nurses involved in KMC implementation. These sources documented implementation experiences, operational adaptations, continuity of care processes, workforce mentoring, family participation and contextual challenges.

Process indicators included KMC initiation among eligible newborns, timing of initiation, continuation of KMC after discharge, completion of scheduled follow-up visits, breastfeeding support provided, family participation in KMC, newborn danger signs and duration of skin-to-skin care. Outcome indicators included breastfeeding status, weight gain and illness episodes.

### Data analysis

Quantitative data were entered into a secure database and analysed using descriptive statistics to summarise KMC initiation, continuation, follow-up coverage, referrals and newborn outcomes.

Qualitative data were analysed thematically to identify barriers, facilitators and operational lessons related to KMC implementation, family engagement, workforce performance and continuity of care. Findings from quantitative and qualitative sources were triangulated to provide an integrated assessment of implementation processes and outcomes.

## Results

The KMC circle of care at PHC Nithauwa began during antenatal care rather than at birth. Antenatal care (ANC) registration remained stable from 2021–2022 to 2023–2024 (n=606, n=623 and n=598 women, respectively), with 151 registrations by June 2024. More importantly, completion of four ANC check-ups increased markedly from 368 women (60.7%) in 2021–2022 to 597 (95.8%) in 2022–2023 and 606 (101.3%) in 2023–24. These ANC contacts enabled frontline staff to introduce newborn care concepts including KMC well before delivery. The same PHC nurses, ANMs and ASHAs who later supported postnatal KMC provided ANC counselling, establishing trust and familiarity that strengthened continuity along the maternal-newborn pathway.

By delivery, many families were already primed for skin-to-skin care and exclusive breastfeeding, making ANC the foundational entry point of the KMC circle of care.

### Birth profile and low birth weight burden

During the implementation period, 1466 births were recorded across the PHC catchment area. Of these, 511 newborns (34.9%) had a birth weight below 2.5 kg, including 17 (1.2%) weighing less than 1.5 kg and 494 (33.7%) weighing between 1.5 kg and 2.5 kg. The proportion of LBW newborns remained consistently high across the 3 years, ranging from 30.4% in 2021–2022 to 39.3% in 2023–2024 (table 1).

**Table 1:** Birth weight distribution in the PHC catchment area

Year	Total births in the catchment area	<1.5 kg	1.5–2.5 kg	>2.5 kg
2021–2022	506	6	148	352
2022–2023	492	5	168	319
2023–2024	468	6	178	284
Total	1466	17	494	955

PHC, primary health centre; PNC, postnatal care; PSBI, possible serious bacterial infection.

### Newborn care coverage and KMC initiation

Between September 2021 and May 2024, 1188 deliveries occurred at PHC Nithauwa, of which 34% (n=409) were LBW (<2.5 kg). 78 cases requiring higher-level care were referred to secondary or tertiary facilities; of these, 17 (4.2%) were babies weighed less than 1.5 kg, all of whom were referred. 15 stillbirths were recorded facility-based provision of KMC demonstrated steady improvement, increasing from 60% coverage in 2021–2022 to 88% in 2023–2024 (table 2). Community-based follow-up extended coverage, with 52% of facility-initiated dyads continuing KMC at home under ANM and ASHA supervision. A minority (16%) of LBW infants did not initiate KMC at the PHC, primarily due to (1) referral to higher-level facilities for complications or (2) early newborn deaths within hours of birth.

**Table 2:** Year-wise births and KMC coverage

Year	Live births (institutional deliveries)	LBW (<2.5 kg)	% LBW	KMC at PHC (n, %)	KMC in community (n, %)
2021–2022	344	107	31	64 (60)	35 (55)
2022–2023	453	141	31	138 (98)	83 (60)
2023–2024	391	161	41	141 (88)	61 (43)
Total	1188	409	34	343 (84)	179 (52)

KMC, Kangaroo Mother Care; LBW, low birth weight; PHC, primary health centres.

### Community follow-up and continuity of care

Among facility-initiated KMC dyads, postnatal follow-up was conducted through structured home visits by ANMs and ASHAs. On average, each mother–infant dyad received six structured postnatal home visits, ensuring continuity between facility and community care. Home visits focused on continuation of skin-to-skin care, exclusive breastfeeding, thermal care, recognition of newborn danger signs and referral support where necessary. ANMs conducted most visits, reinforcing KMC, exclusive breastfeeding, thermal care, recognition of danger signs and timely referrals.

Non-maternal caregivers provided skin-to-skin care in 9.5% of documented dyads. These included grandmothers (n=78), other relatives (n=43), fathers (n=27) and grandfathers (n=4). This ‘shared caregiving’ strengthened household-level continuity, particularly among migratory families and expanded the effective caregiving network within the primary health system.

### Strengthening of core KMC practices

#### Thermal care through skin-to-skin contact

Across 395 recorded mother–infant dyads, SSC duration increased progressively from a mean of 7.3 hours/24 hours in 2021 to 8.2 hours/24 hours in 2022, 10.0 hours/24 hours in 2023, stabilising at 9.0 hours in 2024. The upward trend reflected both behavioural consolidation among mothers and consistent reinforcement by health workers.

#### Feeding practices

Feeding information was available for 395 mother–infant dyads who received KMC and had complete breastfeeding and feeding frequency records during follow-up. Exclusive breastfeeding remained consistently high (>97%) across all years. Mean feeding frequency rose from 6.8 feeds/day in 2021 to 10.6 feeds/day in 2023, stabilising in 2024 (10.1 feeds/day). Regular counselling and household reinforcement by health workers, ANM and ASHA follow-up reflected through enhanced maternal confidence (table 3). Additionally, since this was a rural, primary care setting, a human milk banking facility was not available. In most such cases where direct breastfeeding was not available because mother was very ill, the mother and baby were transferred together to a higher facility. In one case, wet nursing was resorted to, where a family member was lactating. We managed the inversion of nipples and breast engorgement using appropriate evidence-based protocols.

**Table 3:** Exclusive breastfeeding and feeding frequency among KMC dyads

Year	n (dyads)	Exclusive breastfeeding (%)	Mean feeds/24 hours	Median feeds/24 hours
2021	45	97.8	6.78	7
2022	139	98.6	9.66	10
2023	142	98.6	10.63	11
2024	69	98.6	10.10	10
Total	395	98.4	10.05	10

KMC, Kangaroo Mother Care.

### Community monitoring and newborn outcomes

Among 395 dyads followed under community-based KMC, possible serious bacterial infection (PSBI) was identified in only three infants (0.8%), of whom two (67%) were referred to the PHC. Infant survival at follow-up was 99.2%, with two newborn deaths and one loss to follow-up documented during the implementation period. This may reflect inadequate skills in identification of PSBIU by the workers.

Frontline workers documented repeated counselling related to thermal care, breastfeeding, newborn danger signs and referral completion during home visits.

### Findings on implementation processes

#### Continuity and community adaptation

Continuity of KMC practices was maintained despite challenges related to seasonal migration and agricultural labour demands. Flexible scheduling of home visits and involvement of non-maternal caregivers facilitated continuation of skin-to-skin care among some mobile and labour-constrained households.

Counselling during ANC was described by nurses and outreach workers as important in preparing mothers and families for KMC initiation and continuation after delivery. Nurses also reported that repeated counselling and home-based follow-up visits were important in sustaining KMC continuation after discharge. Illustrative examples of continuity support and home-based follow-up are presented in online supplemental appendix 1A and B.

Frontline workers also described gradual improvement in family acceptability of KMC over the implementation period. Nurses and ASHAs reported that some families were initially hesitant because routine clothing practices and daily household work were not always compatible with prolonged skin-to-skin care. Early interactions therefore focused largely on verbal counselling, demonstration and repeated household discussions regarding the purpose and benefits of KMC. Over time, families who observed improvement in infant feeding, warmth and weight gain increasingly accepted the practice and were more willing to continue KMC at home. Nurses reported that this growing familiarity and perceived value of KMC contributed to improved continuation and sustainability of the intervention within communities.

#### Workforce adaptation and mentorship

Continuous field-based mentorship and supportive supervision were described by nurses and supervisors as important for reinforcing KMC practices during routine service delivery. Mentorship activities included joint outreach visits, review meetings and real-time problem-solving during newborn care encounters. Frontline workers reported increasing familiarity with newborn thermal care, breastfeeding counselling, recognition of danger signs and follow-up coordination over the implementation period. An illustrative example is described in online supplemental appendix 1C.

#### Contextual challenges and community engagement

Nurses identified several recurring implementation challenges. These included difficulty sustaining prolonged KMC during nighttime, maternal workload constraints, seasonal migration, inconsistent follow-up among non-catchment populations and reduction in KMC duration once infants appeared clinically stable.

Frontline workers additionally described gender-related differences in family engagement with newborn care in some households, requiring repeated counselling and household-level negotiation to sustain KMC practices. At the same time, nurses and ASHAs reported that some mothers who had positive experiences with KMC later encouraged other women in their villages to adopt skin-to-skin care and exclusive breastfeeding practices for LBW newborns. An illustrative example of community advocacy following KMC experience is provided in online supplemental appendix 1D.

## Discussion

This paper describes how a PHC-community KMC, circle of care, was operationalised within a rural primary healthcare system in southern Rajasthan. The findings suggest that linking antenatal counselling, facility-based KMC initiation, structured home follow-up and community-based newborn monitoring may support continuity of care for LBW and preterm newborns in remote and resource-constrained settings.

The circle of care allowed an integration of newborn care across antenatal, facility and postnatal phases using existing PHC and outreach systems. KMC became embedded within routine maternal–newborn care pathways involving PHC nurses, ANMs, ASHAs and families across facility and household settings rather than functioning as a stand-alone newborn intervention.

### Circle of care as a health systems innovation

The circle of care, anchored in the integration of primary and community systems, emerged as a key health systems innovation in this study. The circle of care is widely recognised as a core attribute of high-quality primary healthcare.^13 14^ Yet in most low-resource settings, care pathways are disrupted by geographical distance, migration, workforce shortages and weak referral linkages.^15^

The KMC circle of care often began during ANC which provided opportunities for introducing families to thermal care, breastfeeding, danger sign recognition and preparation for continuation of KMC after birth. Because the same PHC nurses, ANMs and ASHAs who later supported postnatal KMC also provided antenatal counselling, families frequently encountered a familiar care network across pregnancy, childbirth and postnatal follow-up. This continuity appeared particularly important in a geographically dispersed and migratory setting where fragmentation between facility and community care is common.

Families experienced an ongoing accompaniment by the same cadre of staff**,** which minimised dropouts and reinforced trust. Previous studies from low-resource settings have highlighted that although facility-based KMC initiation may be feasible, continuation after discharge often remains inconsistent because of weak follow-up systems and limited integration between facility and community care. Our findings suggest that integrating KMC into routine outreach and postnatal follow-up pathways may help support continuity beyond the immediate facility period.^4 16^

The integration of PHC and community systems created a horizontal structure of accountability, where outreach workers and PHC nurses shared responsibility for outcomes. The contrast with traditional models is apparent in many rural and tribal health systems; facility care is delivered in isolation, with little assurance of follow-up once the mother and infant return home. By contrast, the closed-loop model we observed ensured that outreach workers and PHC staff shared responsibility for outcomes, creating a horizontal integration between health systems and community-based care. Such integration has been identified as a critical pathway to reducing newborn mortality and strengthening primary healthcare.^17 18^

### Mentorship and workforce adaptation within routine service delivery

Our results also highlight how field-embedded mentorship functioned as both a capacity-building mechanism and an integration strategy. Frontline workers described increasing familiarity with thermal care, breastfeeding support, newborn danger sign recognition and follow-up coordination over time. Structured coordination between PHC nurses, ANMs and ASHAs also appeared to support continuity between facility and community care processes.^19 20^

This circle of care recognised families as co-creators of care, not passive recipients. Family members actively participated in KMC at home, which extended the reach of the health system and enhanced accountability. This co-productive role of families aligns with broader evidence that social participation and lay caregiving can be integral to resilient primary health systems.^19^

This embedding of mentorship and co-creation into everyday practice enabled service delivery to shared stewardship of newborn health.

### Family participation and acceptability of KMC: relational dimensions of integration

The findings additionally suggest that family participation played an important role in the continuation of KMC practices at home. Non-maternal caregivers, including grandmothers, fathers and other relatives, participated in skin-to-skin care in a subset of households, particularly among families affected by migration, agricultural labour demands or maternal workload constraints. Frontline workers also described a gradual improvement in the acceptability of KMC. Over time, families who observed improvement in infant warmth, feeding and weight gain appeared more willing to continue KMC at home and encourage other mothers to adopt similar practices.

Families increasingly reported seeing PHC staff as partners rather than distant providers**,** a shift that is particularly meaningful in rural and tribal settings where public health services are often perceived as detached and inaccessible.^21^ Trust, in turn, has been shown to influence adherence, help-seeking behaviour and community participation in health interventions.^22^ These findings highlight the need for repeated counselling, family engagement and community-level adaptation in sustaining KMC practices at home.

### Implications for policy and practice

This study reinforces the argument that strengthening newborn care requires deliberate integration of primary and community systems, creating a functional circle of care that includes families as co-creators. Three key implications emerge. First, the circle of care must be deliberately designed into service delivery models. This requires moving beyond episodic, facility-bound approaches toward horizontally integrated systems that link households, outreach workers and PHC teams. Second, mentorship-based capacity building should be institutionalised within PHC to ensure technical fidelity and shared accountability. Third, interventions must anticipate and adapt to contextual barriers, including migration, gender norms and caregiver fatigue. Without such adaptations, continuity risks becoming uneven and reinforcing inequities.

### Strengths, limitations and future directions

The study’s strengths include its real-world implementation design, longitudinal follow-up and integration across facility and community levels. Limitations include reliance on retrospectively reviewed routine implementation records. Second, gestational age stratification were inconsistently documented for some newborns, limiting subgroup analysis by prematurity category. Third, the implementation was conducted within a single PHC setting and did not include a comparison group. Fourth, several implementation observations were derived from field documentation and in-depth interviews conducted for operational learning rather than formal qualitative research.

Despite these limitations, the paper provides insights into how continuity-oriented newborn care pathways may be integrated within rural PHC systems in low-resource settings. Future work should further examine implementation approaches that strengthen continuity across antenatal, facility and postnatal care while addressing migration, referral coordination and long-term community follow-up.

## Supplementary material

10.1136/bmjpo-2026-004568 online supplemental appendix 1

## Data Availability

Data are available upon reasonable request.
