# Supplementary material for: Circle of care model for strengthening newborn care through Kangaroo Mother Care integration in primary health systems in rural India
Source: BMJ Paediatr Open. 2026 Sep 3;10(1):e004568. doi: 10.1136/bmjpo-2026-004568 (PMC13548244; doi:10.1136/bmjpo-2026-004568)
Supplement: Supplementary file 1 [file bmjpo-2026-004568.reviewer_comments.pdf]

PEER REVIEW HISTORY

BMJ Paediatrics Open publishes all reviews undertaken for accepted manuscripts. Reviewers are asked to complete a checklist review form and are provided with free text boxes to elaborate on their assessment. These free text comments are reproduced below.

ARTICLE DETAILS

**TITLE (PROVISIONAL)**	A Circle of Care Model for Strengthening Newborn Care through Kangaroo Mother Care Integration in Primary Health Systems in Rural India
**AUTHORS**	Thakral, Yogita<br>Gulechha, Ashmita<br>Mohan, Sanjana<br>Mohan, Pavitra

VERSION 1 – REVIEW

**REVIEWER NAME**	Nanavati, Ruchi
**REVIEWER AFFILIATION**	Seth GS Medical College and KEM Hospital, Neonatology
**REVIEWER CONFLICT OF INTEREST**	No Conflict of Interest
**AI DISCLOSURE**	No, I have not used AI
**DATE REVIEW RETURNED**	12-Mar-2026

**GENERAL COMMENTS**	I wish to congratulate authors for conducting the need of time study in KMC.<br><br>It is mentioned that 34% are LBW. Further division as per weight (HRLBW, VLBW, ELBW) and gestational age (late/ moderate/ early preterm) is necessary as they determine outcomes (morbidity and mortality) and quality of survival.<br><br>No KMC in 16%. Please provide further details as why?<br><br>Exclusive breastfeeding rate is very high and appreciated. But its challenging if the cohort includes VLBW, ELBW, or moderate/ early preterm infants. So details are required.<br>Also mention what was planned in case of breastfeeding not possible.<br>Also mention the duration of exclusive breastfeeding.<br>Please mention about Human Milk Bank facility.<br><br>The concept and implementation of "circle of care" is well appreciated.

**REVIEWER NAME**	Rao, Suman P. N.
**REVIEWER AFFILIATION**	
**REVIEWER CONFLICT OF INTEREST**	No Conflict of Interest
**AI DISCLOSURE**	I used the AI to correct the grammar and make the rejection soft.<br><br>The research paper was not uploaded but my reviewer comments (which are given below) were uploaded on GEMINI AI. The gemini

	output was reviewed and revised<br><br>The paper is a good narrative of an innovative way of delivering essential newborn care at the primary care level leveraging KMC. It highlights how embedding KMC improves workforce capacity, strengthens systems and improves mortality. It is excellent as an implementation report. However, there are several aspects which need clarification.<br>1. It is not clear what are the primary and secondary outcomes and how they were assessed. How can we conclude that the capacity of the staff improved or systems were strengthened?<br>2. While mortality is low, there is no comparison to show that survival has improved.<br>3. It is stated that families were "co-creators" of newborn care. How was this assessed?<br>4. One of the major limitations of this study is that it was done only in ONE PHC. (a very small area)<br>5. It is mentioned on page 6, that BHS "managed" staffing. What does it mean?<br>6. Is it routine for ANMS to conduct home visits or was it done only in this project.<br>7. Why was KMC started within few hours and not in the first hour as per the recommendation?<br>8. In the results section, referring to increase in 4 ANC check ups, percentages may be more meaningful than numbers.<br>9. How was KMC hours documented, how accurate was it at home. and the hours mentioned in the paper... is it in this in hospital or at home or both?<br>10. It is stated that mothers had "enhanced confidence" How was it assessed?<br>11. In table 2, n is a subset of the total population, how was this subset chosen and why?<br>12. Considerable section of the results (3.4 onvwards) reads more like discussion rather than results. If this is from the quali segment of the research, its analysis plan is not clear, and there are no direct quotes.<br>13. On page 10 , it is mentioned that 70% with danger signs were traiges. How was this data collected and analysed.<br>14. Is there any documentation of the live encounter with the mentors (page 11)<br>15. It is stated that the learning-doing model improved confidence, accountability and technical proficiency.. how was this assessed?<br>16. It is written that the FLWs 'expanded" their role to include "counseling" which is actually an integral part of the work.<br>17. The two anecdotal case reports mentioned are nice to read but do not add ot the reuslts.<br>18. Was there a tool to assess family's perception?<br>19. Is data available to show how many got ANC counseling on KMC and how it improved over time?<br>20. In the figure on circle of care, ANC is not included.<br>21. Some references are inappropriate. Eg Ref 9 should refer to the NEJM iKMC trial.. But it refers to a review article. Similarly, the community and home based KMC studies were done BEFORE the ikmc trial and not subsequently.
**DATE REVIEW RETURNED**	10-May-2026

**GENERAL COMMENTS**	General Comments<br>The authors present a commendable narrative on an innovative approach to delivering essential newborn care (ENC) at the primary

	care level by leveraging KMC. The manuscript highlights the potential for KMC to improve workforce capacity and strengthen health systems. While the paper serves as an excellent implementation report and provides valuable insights into local adaptation, the methodological rigor and comparative data are not optimal. At this stage, the manuscript is primarily descriptive, and several critical areas require substantiation.<br>Major Concerns<br>1. Methodological Framework and Outcome Assessment<br>The primary and secondary outcomes are not clearly defined, nor is the methodology used to assess them. To support the conclusion that staff capacity improved or systems were strengthened, the authors could provide objective metrics or validated assessment tools used for these evaluations. Similarly, claims regarding "enhanced confidence," "accountability," and "technical proficiency" (page 11) appear subjective without a clear description of the evaluation framework or pre- and post-intervention data. Documentation and quantification of processes such as live encounters are missing.<br><br>2. Evidence of Impact and Generalizability<br>While the reported low mortality is encouraging, the lack of a comparison group or historical baseline makes it difficult to attribute these outcomes directly to the intervention. Furthermore, the study's scope is limited to a single Primary Health Center (PHC). This small sample size and localized context significantly limit the generalizability of the findings.<br>3. Qualitative Analysis and Data Presentation<br>A significant portion of the results (from section 3.4 onwards) reads as a discussion rather than a presentation of data. If these findings are derived from a qualitative research segment, the analysis plan (e.g., thematic analysis) could be explicitly stated, and the findings supported by direct quotes from participants. Additionally, the term "co-creators" regarding families is used frequently, but the manuscript does not explain the criteria or methodology used to evaluate this level of engagement.<br><br>4. Data Accuracy and Documentation<br>There are several points where data collection methods remain unclear:<br><br>How were KMC hours documented in the facility and at home, and how was this accuracy verified?<br><br>The triage data regarding danger signs (70% on page 10) requires a description of the data collection and analysis process.<br><br>In Table 2, the subset 'n' is not clearly defined in relation to the total population; the selection criteria for this subset should be clarified.<br><br>Minor and Technical Points<br>Clinical Protocols: The authors state KMC was initiated within a "few hours." Please clarify why this did not align with the current recommendation of initiation within the first hour.<br><br>Operational Definitions: Clarify what is meant by BHS "managing" staffing (page 6). Additionally, clarify if ANM home visits were a project-specific intervention or part of routine care.<br><br>Role Definitions: The manuscript suggests FLWs "expanded" their

	role to include counseling; however, counseling is typically considered an integral part of their existing duties.<br><br>Formatting: In the results section regarding ANC check-ups, please provide percentages rather than raw numbers to offer better context for the reader.<br><br>Visual Aids: The "Circle of Care" figure should ideally include ANC to provide a complete picture of the continuum of care.<br><br>Case Reports: While the anecdotal case reports are illustrative, they do not currently add substantive weight to the results and may be better suited for a supplementary section.<br><br>References and Citations<br>A thorough review of the references is required. Specifically:<br><br>Reference 9: This should cite the definitive NEJM iKMC trial rather than a review article.<br><br>Chronology: Ensure the text accurately reflects the timeline of KMC research; several community and home-based studies cited as "subsequent" were actually conducted prior to the iKMC trial.<br><br>Final Recommendation:<br>While the project itself is of great importance and provides a "real-world" look at KMC implementation, the manuscript lacks the comparative analysis and methodological depth of aa research article. I encourage the authors to strengthen the data analysis and consider resubmitting as an implementation report rather than a research article.

VERSION 1 – AUTHOR RESPONSE

Response to the Reviewers
Manuscript Title: A Circle of Care Model for Strengthening Newborn Care through Kangaroo Mother Care Integration in Primary Health Systems in Rural India
We thank the Associate Editor and Reviewers for their inputs and constructive feedback. We appreciate the opportunity to revise the manuscript and have undertaken revisions to improve methodological clarity, strengthen the implementation framework, and enhance transparency regarding intervention delivery, monitoring, and outcome assessment as indicated in the detailed review.
However, we were unable to identify a specific submission category titled "Implementation Report" within the available article categories of BMJ Paediatrics Open. We have therefore revised the manuscript to align more closely with the expectations of an implementation-focused paper and respectfully leave it to the discretion of the Editor and Reviewers to determine the most appropriate category under which the manuscript may be considered.
A point-by-point response to all comments is provided below. Our responses are highlighted in blue.
Response to the Associate Editor
The study is stated to be an implementation research. However, there is not sufficient description of how the initial model of implementation was developed and what iterative processes were used to achieve the desired coverage and its quality.
We agree and have revised the Methods section.
A new subsection, “Development of the Integrated Newborn Care Model,” has been added describing the formative phase conducted between April and August 2021. This phase involved consultations with PHC nurses, ANMs, ASHAs, supervisors, and mothers accessing antenatal and postnatal

services. These consultations identified barriers to continuity of newborn care, including limited familiarity with prolonged skin-to-skin care, weak facility-community coordination, geographic barriers, seasonal migration, and challenges in follow-up of vulnerable newborns.

We also describe the iterative implementation processes used during the implementation, including supervisory reviews, mentoring visits, monthly review meetings, case discussions, refinement of counselling approaches, adaptation of follow-up schedules, strengthening referral pathways, and improvements in documentation systems.

There is no information on the target population in the PHC area that were eligible for the interventions described in the study and what proportion received the desired intervention and its quality.

We have added this under the intervention sub section in the methods section

The revised manuscript now clearly defines the target population, including low birth weight newborns (<2500 g), clinically stable preterm infants, and newborns identified during community outreach who required KMC initiation or continuation.

A new Results subsection describing the birth profile of the PHC catchment area has also been added, including total births, low birth weight prevalence, birth-weight distribution, institutional deliveries, referrals, and stillbirths. Coverage indicators are now presented using clearly defined denominators to enable interpretation of intervention reach.

Did the study have any process indicators? If yes, it would be desirable to describe them.

We have added a new subsection entitled “Data Sources, Process Indicators and Outcome measures in the methods section.

Who measured the outcome indicators? How were they measured and at what frequency?

The Methods section has been revised to clarify data collection procedures.

Facility-based indicators were recorded by PHC nurses during admission, delivery, postnatal observation, and discharge. Community-level indicators were documented by ANMs and ASHAs during scheduled home visits. Supervisory staff reviewed records during routine monitoring visits. Measurements were conducted during facility stay, on days 3 and 7 after discharge, and weekly thereafter up to eight weeks, with additional assessments undertaken when danger signs were identified.

Similarly how was the quality of intervention delivery assessed and by whom?

This is added to the intervention section. Implementation quality was assessed through direct observation of KMC practices, review of counselling sessions, verification of follow-up records, supervisory visits, and monthly review meetings. Assessments were undertaken by senior nursing supervisors, program coordinators, and PHC leadership teams.

Quality indicators included correct KMC positioning, breastfeeding support practices, completion of scheduled follow-up visits, documentation quality, and appropriate referral of newborn danger signs.

Was there a formative phase of the study prior to rolling out the intervention?

We have now explicitly described the formative phase undertaken prior to implementation. This phase included stakeholder consultations, assessment of newborn care gaps, review of referral patterns, workforce capacity assessment, and development of follow-up and referral pathways. Findings informed the improvement of the integrated KMC model overtime.

Sections 3.4, 3.5, 3.6 in the Results are better described in the Methods as they are probably more reflective of processes.

We agree and have reorganized the manuscript accordingly.

Descriptions of mentoring systems, supervision mechanisms, referral pathways, community engagement processes, and implementation adaptations have been moved from the Results section into the intervention subsection of the Methods section. The Results section now focuses primarily on implementation coverage, process indicators, and newborn outcomes.

The method section needs to be described in greater detail and articulated better for the reader to understand the process with greater clarity and also enable replication at another site if one so desires.

The revised structure now includes: Study Setting, Development of the Integrated Newborn Care

Model, Intervention, Data Sources, Process Indicators and Outcome Measures, Data Analysis These revisions provide details regarding implementation processes, workforce preparation, supervision systems, follow-up procedures, outcome measurement, and quality assurance mechanisms.

Response to Reviewer 1

It is mentioned that 34% are LBW. Further division as per weight (HRLBW, VLBW, ELBW) and gestational age (late/moderate/early preterm) is necessary.

We have added a more detailed birth-weight distribution among low birth weight newborns. However, gestational age was not consistently recorded in routine program records throughout the implementation period. We have acknowledged this limitation in the Discussion

No KMC in 16%. Please provide further details as why?

We have clarified this in the Results section. A minority (16%) of LBW infants did not initiate KMC at the PHC, primarily due to (i) referral to higher-level facilities for complications, or (ii) early newborn deaths within hours of birth.

Exclusive breastfeeding rate is very high. Further details are required.

We have clarified that the majority of newborns receiving KMC were clinically stable LBW infants. Very low birth weight newborns and clinically unstable infants were generally referred to higher-level facilities according to routine referral protocols.

Please mention what was planned in case breastfeeding was not possible and mention the Human Milk Bank facility.

We have clarified that no Human Milk Bank facility was available at the PHC. When direct breastfeeding was temporarily not possible, mothers were supported with breast milk expression and feeding assistance. Newborns requiring advanced feeding support were referred to higher-level facilities.

The concept and implementation of “Circle of Care” is well appreciated.

We thank the reviewer for this encouraging feedback.

Response to Reviewer 2

Major Concern 1: Methodological Framework and Outcome Assessment

We have strengthened the methodological framework as described above.

The revised manuscript now clearly distinguishes process indicators and outcome indicators and describes how these were measured. We have also revised the manuscript to present the observations as qualitative implementation findings rather than measured outcomes as appendices.

Major Concern 2: Evidence of Impact and Generalizability

The manuscript has been repositioned as an implementation assessment using routine program data rather than an effectiveness study. We now explicitly acknowledge the absence of a comparison group, lack of baseline measurements, and the limitations associated with a single-site implementation in the limitations section.

Major Concern 3: Qualitative Analysis and Data Presentation

We have added the qualitative part and case stories as appendices now through a reference in the sub-section called ‘Findings on Implementation Process’ in the results section. The idea of cocreation is explained in further detail as indicated by the reviewer: This circle of care recognized families as co-creators of care, not passive recipients. Family members actively participated in KMC at home, which extended the reach of the health system and enhanced accountability. This co-productive role of families aligns with broader evidence that social participation and lay caregiving can be integral to resilient primary health systems (19).

This embedding of mentorship and co-creation into everyday practice enabled service delivery to shared stewardship of newborn health.

Major Concern 4: Data Accuracy and Documentation

The Methods section now clarifies that KMC duration was documented through facility records during admission and caregiver reporting during community follow-up visits. We acknowledge the limitations of self-reported home-based KMC duration in the Discussion.

Additional details have been provided regarding the identification and recording of newborn danger signs, referral processes, and the denominators used in Table 3 (earlier Table 2 in the first version

submitted).
Minor Comments
We have addressed all minor comments, including clarification of KMC initiation timing, the role of BHS in staffing and supervision, the routine nature of ANM home visits, use of percentages in reporting, revision of the Circle of Care figure to include antenatal care, relocation of case examples to supplementary material, and updating references to include the NEJM Immediate KMC trial while revising the chronology of KMC literature.

VERSION 2 – REVIEW

**REVIEWER NAME**	Nanavati, Ruchi
**REVIEWER AFFILIATION**	Seth GS Medical College and KEM Hospital, Neonatology
**REVIEWER CONFLICT OF INTEREST**	No Conflict of Interest
**AI DISCLOSURE**	No, I have not used AI
**DATE REVIEW RETURNED**	06-Jul-2026

**GENERAL COMMENTS**	The authors have revised the entire manuscript well.<br>HMB facility was not available and when direct breastfeeding was not temporarily possible, ...Please mention what was done in this scenario.<br>Also mention % of infants transferred to higher centres to get correct idea as exclusive BF rates are very high.<br>It is mentioned that newborns requiring advanced feeding support were transferred to higher centres. Please give numbers (%).

VERSION 2 – AUTHOR RESPONSE

Reviewer: 1
Ruchi Nanavati, Seth GS Medical College and KEM Hospital
Comments to the Author
The authors have revised the entire manuscript well.
We thank the reviewers for their earlier comments which helped in revision of the manuscript.

1. HMB facility was not available and when direct breastfeeding was not temporarily possible, ...Please mention what was done in this scenario.
Response: Since this was a rural, primary care setting, Human milk banking facility was not available. In most such cases where direct breastfeeding was not available because mother was very ill, the mother and baby were transferred together to a higher facility. In one case, wet nursing was resorted to, where a family member was lactating. We managed the inversion of nipples and breast engorgement using appropriate evidence based protocols.
2. Also mention % of infants transferred to higher centres to get correct idea as exclusive BF rates are very high.
Response: Among all institutional births (1188). Overall, 78 babies were referred out of 1178 (6.6%) births. Of all live births, 409(34%) were low birth weight. Of these 17 (4.2%) were babies weighed less than 1.5 kg, all of whom were referred.
BF rates appear to be high because of the following reasons:
a. This was predominantly a tribal community with 83.55% of the tribal population in the PHC catchment area. Among tribal communities, exclusive breastfeeding rates are much higher than in non-tribal communities (Reddy N et al., 2023)
b. As mentioned above, babies less than 1.5 kg were referred to a higher facility. Only those babies more than 1.5 kg were provided the intervention. Besides babies with complications such as birth asphyxia and sepsis were referred to a higher facility and were not included in our sample. So the

babies who were sick and were very small were excluded and therefore likely to be exclusively breastfed.
c. Circle of care described in the paper helped mothers establish and continue breastfeeding in normal weight and low weigh babies.

3. It is mentioned that newborns requiring advanced feeding support were transferred to higher centres. Please give numbers (%).
Response: As mentioned in response to query number 2, 78 babies were referred, out of which 17 were because of very low birth weight (<1500 gms). We did not capture the reasons for referral for each of them, but anecdotally, we know that atleast one mother and baby was referred specifically for advanced feeding support
Reference
Reddy N, S., Dharmaraj, A., Jacob, J., & Sindhu, K. N. (2023). Exclusive breastfeeding practices and its determinants in Indian infants: Findings from the National Family Health Surveys-4 and 5. International Breastfeeding Journal, 18, 69. https://doi.org/10.1186/s13006-023-00602-z
