# Supplementary material for: Circle of care model for strengthening newborn care through Kangaroo Mother Care integration in primary health systems in rural India
Source: BMJ Paediatr Open. 2026 Sep 3;10(1):e004568. doi: 10.1136/bmjpo-2026-004568 (PMC13548244; doi:10.1136/bmjpo-2026-004568)
Supplement: Supplementary file 2 [file bmjpo-10-1-s001.docx]

**Appendix 1. Illustrative Implementation Vignettes from the KMC Circle of Care**

**Appendix 1A. Continuity of Follow-up Beyond the Routine PHC Catchment Area**

A 1600 g low birth weight newborn residing outside the routine PHC catchment area required continued post-discharge KMC follow-up after facility initiation. Because the family lived in a geographically difficult and migratory area, routine follow-up risked discontinuation. A male outreach nurse familiar with the local area coordinated with the PHC ANM to conduct repeated home visits, monitor infant weight gain, reinforce breastfeeding and skin-to-skin care, and support continuation of KMC within the household. This case illustrated how local outreach coordination and flexible follow-up arrangements were used to maintain continuity of newborn care beyond administrative catchment boundaries.

**Appendix 1B. Home-Based Continuation of KMC and Follow-up Support**

Frontline workers described repeated counselling and home-based follow-up as important for sustaining continuation of KMC after discharge from the PHC. In one documented instance, a 2.45 kg newborn whose mother continued KMC practices with regular home-based support demonstrated progressive weight gain to 3.45 kg by day 28 of follow-up. Nurses and outreach workers reported that repeated counselling visits, breastfeeding support, and involvement of family caregivers were important in maintaining adherence to KMC practices during the postnatal period.

**Appendix 1C. Operational Challenges During Community-Based KMC Follow-up**

Nurses identified several recurring operational challenges during the implementation of community-based KMC. Mothers frequently reported difficulty sustaining prolonged skin-to-skin care during nighttime because of fatigue and concerns regarding infant safety. Frontline workers also observed that some families reduced KMC duration once infants appeared clinically stable, requiring repeated counselling reinforcement during follow-up visits. Seasonal migration and agricultural labour demands additionally affected continuity of follow-up among some households, particularly in geographically remote villages.

**Appendix 1D. Community Advocacy Following Positive KMC Experience**

Frontline workers described instances in which mothers who had positive experiences with KMC subsequently encouraged other families within their villages to adopt skin-to-skin care and exclusive breastfeeding practices for low birth weight newborns. In one such case, a mother whose low birth weight infant demonstrated steady improvement following continued KMC and home-based follow-up later began sharing her experience informally with pregnant women and other mothers during village interactions and antenatal meetings. Nurses and ASHAs reported that such peer-led encouragement sometimes increased community familiarity with KMC practices and reduced hesitation among families encountering KMC for the first time.
